# Healthcare providers’ perspectives on headache care in Azerbaijan: results of a national cross-sectional survey

**DOI:** 10.3389/fneur.2026.1886668

**Published:** 2026-09-04

**Authors:** Sarkhan Amirguliyev, Aynur Özge, Derya Uludüz, Semih Taşdelen, Tayyar Şaşmaz, Rami Burstein

**Affiliations:** 1 Migraine and Headache Specialist, Azerbaijan State Institute of Advanced Training of Doctors named after Aziz Aliyev, Baku, Azerbaijan; 2 Department of Neurology, Mersin University, School of Medicine, Mersin, Türkiye; 3 Türkiye and NOROM Neuroscience and Excellence Center, Ankara, Türkiye; 4 Department of Neurology, Atlas University Medical Faculty, İstanbul, Türkiye; 5 Department of Neurology, İstanbul Faculty of Medicine, İstanbul University, İstanbul, Türkiye; 6 Department of Public Health, Mersin University, School of Medicine, Mersin, Türkiye; 7 John Hedley-Whyte Professor of Anesthesia and Neuroscience, Harvard Medical School, Vice Chairman Neuroscience Department of Anesthesia and Critical Care, Beth Israel Deaconess Medical Center, Boston, MA, United States

**Keywords:** Azerbaijan, drug therapy, headache disorders, health services accessibility, health workforce, low- and middle-income countries, low- and middle-income countries (LMICs) headache, migraine

## Abstract

**Background:**

Headache disorders are prevalent in Azerbaijan, yet data on system-level care delivery, treatment options, and provider preparedness remain scarce. Using a national, cross-sectional survey of healthcare professionals, this study aimed to assess the current state of headache care in Azerbaijan.

**Methods:**

A national, cross-sectional survey was conducted using a convenience sampling approach, targeting healthcare professionals involved in headache management across Azerbaijan. Data were collected between December 2024 and May 2025, 333 healthcare professionals, including neurologists (25.9%), general practitioners (27.9%), pharmacists (21.9%), and pediatricians (24.3%). Data were collected using a modified HARDSHIP-based questionnaire that addressed provider training, access to medications, treatment practices, access to migraine-specific resources, and perceptions of patient demographics. Descriptive statistics and comparative analyses were performed to describe regional and professional disparities.

**Results:**

All participating healthcare professionals reported receiving some form of training in headache care. Within the survey sample, respondents were predominantly based in urban hospital settings, whereas relatively few practiced in rural areas and none reported working in community-based healthcare centers. These findings suggest possible geographic differences in the distribution of participating healthcare professionals; however, because the survey was based on convenience sampling, they should be interpreted as descriptive observations rather than evidence of nationally representative workforce disparities. The most frequently reported acute treatments were NSAIDs (67.3%) and paracetamol (27.6%). The most commonly reported preventive medications were amitriptyline (75.7%), magnesium (51.1%), gabapentin (24.6%), and valproate (24.3%). Migraine-specific therapies such as triptans were reported infrequently (15.3%), whereas beta-blockers were not reported to be used for migraine prevention. Newer migraine-specific therapies, including anti-CGRP monoclonal antibodies and gepants, were not available within the national healthcare system at the time of data collection.

**Conclusion:**

This national provider-based survey provides the first perception-based overview of headache care in Azerbaijan and identifies priorities for future health-service planning. Although participating healthcare professionals reported exposure to headache-related training and access to commonly used medications, the findings suggest potential geographic differences in provider distribution and limited use of migraine-specific therapies. Because the survey was based on healthcare providers’ perceptions derived from a convenience sample, the findings should be interpreted as complementary to, rather than substitutes for, patient-level or administrative health-system data. Future nationally representative studies are needed to guide the development of structured, evidence-based headache services.

## Introduction

Headache disorders—particularly migraine—are among the most prevalent and disabling neurological conditions worldwide. Global burden analyses consistently rank headache disorders among the leading causes of years lived with disability (YLDs), with migraine alone accounting for a substantial proportion of non-fatal disease burden across all age groups (1, 2). Beyond individual suffering, migraine imposes considerable societal and economic costs through reduced productivity, absenteeism, presenteeism, and increased healthcare utilization (3).

The impact of headache disorders is disproportionately amplified in low- and middle-income countries (LMICs), where limitations in healthcare infrastructure, specialist workforce capacity, and access to evidence-based therapies contribute to underdiagnosis and undertreatment (3, 4). In such settings, fragmented care pathways and reliance on non-specific analgesics are common, increasing the risk of avoidable disability and headache chronification (5, 6).

The International Headache Society (IHS), in collaboration with regional networks such as the Middle East, North Africa, and Asia (MENAA) Headache Group, has called for the implementation of structured headache services and quality care indicators to improve diagnosis, management, and equity in headache care globally (1, 2). Structured headache services refer to organized, tiered models of care that integrate headache management across primary, secondary, and tertiary healthcare levels, with clearly defined referral pathways, standardized diagnostic approaches, and quality indicators (5, 7). Evidence from multiple regions demonstrates that such models improve diagnostic accuracy, rational medication use, cost-effectiveness, and equity of access—particularly in health systems with limited specialist availability (2, 7).

Azerbaijan represents a compelling case for examining these issues. Azerbaijan operates a mixed public healthcare system that has undergone substantial reforms in recent years, including the progressive implementation of mandatory health insurance and efforts to strengthen primary healthcare services. Despite these developments, access to specialized neurological care remains concentrated in urban centers, and structured headache services have not yet been systematically integrated into routine healthcare delivery. Available epidemiological data indicate that primary headache disorders affect approximately 18% of the population in Azerbaijan, with migraine representing one of the most common neurological disorders (8). This is broadly consistent with estimates from the MENA region. While headache prevalence data from Azerbaijan have been reported in selected populations (8), system-level evidence describing how headache care is delivered in routine clinical practice remains limited. There is a lack of data on provider-side factors such as training exposure, medication availability, prescribing practices, and perceived structural barriers—elements that are critical for service planning and policy development (9, 10).

This current study presents findings from a national survey conducted as part of the IHS/MENAA initiative to evaluate the state of headache care in Azerbaijan. By focusing on healthcare professionals involved in headache management, the study explores provider distribution across healthcare settings, exposure to headache-related training, availability and use of acute and preventive medications, and perceived barriers within the healthcare system. By analysing healthcare provider characteristics, training levels, treatment availability, and patient access to medications, the study aims to:

- Describe geographic patternsin headache care between urban and rural regions.
- Evaluate the adequacy of provider training in diagnosing and managing migraine.
- Examine economic and systemic barriers to effective headache treatment.

Together, these findings aim to provide the first country-specific description of healthcare providers’ perspectives on headache care in Azerbaijan, thereby establishing a foundation for future health-services research and policy development (1, 2, 6). To the best of our knowledge, this is the first nationwide survey to evaluate healthcare providers’ perspectives on headache care capacity, provider preparedness, medication availability, and perceived barriers to headache service delivery in Azerbaijan. Unlike previous studies that primarily described headache prevalence, the present study focuses on healthcare system capacity and provider-reported aspects of headache care. By identifying current strengths and gaps in headache care delivery, the present survey is intended to provide evidence that may inform future educational initiatives, health-service planning, and policy recommendations aimed at improving equitable access to evidence-based headache care in Azerbaijan.

## Methods

### Study design and setting

This research was conducted as part of a broader multinational initiative led by the International Headache Society (IHS) in collaboration with the Middle East, North Africa, and Asia (MENAA) Headache Group. The study was designed as a national, cross-sectional survey and aimed to assess healthcare provider–reported practices, training exposure, medication availability, and perceived system-level barriers related to headache care in Azerbaijan. Data were collected using an adapted version of the Headache-Attributed Restriction, Disability, Social Handicap, and Impaired Participation (HARDSHIP) questionnaire. The HARDSHIP instrument was developed by the Global Campaign against Headache and has been widely used internationally to assess headache-related burden and healthcare impact (10). The original questionnaire comprises multiple sections covering headache characteristics, disability, healthcare utilization, and social impact and typically takes approximately 20–30 min to complete.

For the purposes of the present study, the questionnaire was modified to focus on healthcare provider perspectives rather than patient-reported outcomes. The adapted version was developed through multidisciplinary expert review to ensure content relevance, linguistic clarity, and contextual suitability for the Azerbaijani healthcare system. In addition, a formal linguistic and psychometric validation study of the adapted questionnaire has been completed independently and is currently under peer review. Because that validation study is being considered for separate publication, its detailed reliability and validity results are not presented in the current manuscript. The study was conducted across multiple cities and regions in Azerbaijan, ensuring a representative sample of both urban (e.g., Baku, Ganja) and rural (e.g., Shaki, Lankaran) populations. This approach allowed for a more comprehensive evaluation of headache burden and disparities in healthcare access across diverse socioeconomic and healthcare settings.

This manuscript is reported in accordance with the Strengthening the Reporting of Observational Studies in Epidemiology (STROBE) Statement for cross-sectional studies.

### Participant recruitment and data collection

The study targeted healthcare professionals involved in diagnosing, treating, or dispensing medications for headache disorders, including neurologists, general practitioners, pediatricians, and pharmacists. No patients were directly enrolled or surveyed, and all patient-related information reflects healthcare providers’ estimates based on their routine clinical experience.

1. Healthcare providers involved in headache diagnosis and management include neurologists, general practitioners, pharmacists, and other healthcare professionals. A total of 333 healthcare providers from Azerbaijan participated in the survey.
2. Patients with headache disorders, assessed through physician-reported data and self-reported symptoms.

To maximize geographic coverage, members of the IHS/MENA-A collaborative network and regional professional contacts were asked to disseminate the survey within their respective administrative regions whenever possible. This approach was intended to encourage participation from healthcare professionals practicing in different parts of Azerbaijan rather than to obtain a proportionally representative sample of the national healthcare workforce. An open-invitation approach was used, and the survey was administered electronically via Google Forms (Google LLC, Mountain View, CA, USA).

Healthcare professionals within the established professional networks were invited to participate through these dissemination channels. Because the survey was distributed via open professional networks, an exact response rate could not be calculated. A total of 360 questionnaires were submitted during the study period. Because the survey was disseminated through open professional networks, institutional mailing lists, and regional contacts, the total number of eligible healthcare professionals who received the invitation could not be determined. Therefore, a conventional response rate could not be calculated. After data validation and completeness checks, 27 questionnaires with incomplete responses (missing data) were excluded, and 333 questionnaires were included in the final analysis. Data collection was conducted between December 2024 and May 2025.

### Preparatory procedures

Before initiating data collection, virtual preparatory meetings were conducted with local investigators and representatives in Azerbaijan. The purpose of these meetings was to (i) ensure cultural and health-system appropriateness of the adapted questionnaire, (ii) standardize survey dissemination procedures across regions, and (iii) harmonize ethical and informed consent procedures in line with the overarching IHS/MENAA study framework.

Adapted HARDSHIP-based questionnaire used for the national survey of healthcare professionals in Azerbaijan. Additional sections on medication affordability, availability, and healthcare facility distribution were included to enhance the study’s relevance for future health policy recommendations. The adapted questionnaire was not developed as a psychometric scale intended to generate composite scores; rather, it served as a structured data-collection instrument for descriptive health-services research.

### Survey components and variables

The adapted questionnaire included sections on:

- Demographics of healthcare providers (age, gender, education level, years of practice, healthcare setting)
- Healthcare providers’ estimates of headache frequency, burden, and patient characteristics encountered in routine clinical practice. Healthcare system factors (access to medications, affordability, insurance coverage)
- Diagnosis and treatment patterns (commonly prescribed medications, non-pharmacological interventions)
- Barriers to headache care (availability of neurologists, treatment costs, patient awareness, and education)

### Data management and statistical analysis

Because this study was designed as an exploratory national cross-sectional survey using convenience sampling, no formal *a priori* sample size calculation was performed. Rather than testing a predefined hypothesis, the objective was to obtain the broadest feasible national representation of healthcare professionals involved in headache care across Azerbaijan. Therefore, all eligible responses received during the study period were included after data validation and completeness checks. Survey data were compiled and analyzed using SPSS Statistics 26.0. Descriptive statistics summarized demographic characteristics, headache prevalence, and patterns of healthcare utilization. Continuous variables were reported as mean ± standard deviation (SD), and categorical variables as frequencies and percentages. Data normality was assessed using the Shapiro–Wilk test together with visual inspection of histograms and Q–Q plots. Because this study used a convenience sampling design and was intended as descriptive health-services research, all statistical comparisons should be interpreted as exploratory rather than confirmatory. The reported *p*-values are presented only to describe observed patterns within the study sample and should not be interpreted as evidence of population-level differences. Accordingly, no adjustment for multiple comparisons was performed. All patient-related percentages reported in this study (e.g., socioeconomic status, educational level, age distribution, and medication affordability) represent healthcare providers’ aggregated estimates based on their routine clinical experience rather than directly measured patient-level data. Accordingly, these values should not be interpreted as population-based prevalence estimates.

Comparative analyses assessed differences in headache burden and healthcare access between urban and rural populations. Chi-square tests were applied for categorical variables, while independent *t*-tests or the Mann–Whitney *U* test were used for continuous variables, depending on the data distribution. A *p*-value of less than 0.05 was considered statistically significant. Because the primary objective of this study was descriptive and exploratory, no formal adjustment for multiple comparisons was applied. The reported *p*-values are presented to facilitate interpretation of observed patterns within the study sample and should therefore be interpreted cautiously as exploratory rather than confirmatory findings.

### Ethical considerations

Ethical approval was obtained from the Institutional Review Board of Toros University, Mersin, Türkiye on behalf of the coordinating the IHS/MENAA initiative (Approval No: 24.12.2024/218). The study included only licensed healthcare professionals and involved no patients or vulnerable populations. Participants were recruited through open professional networks and received a standardized information statement prior to participation. Verbal informed consent was obtained by local investigators using an IRB-approved script; consent was documented by participants’ voluntary initiation and completion of the anonymous electronic survey, which served as confirmation of informed consent.

## Results

### Characteristics and geographic distribution of healthcare professionals

A total of 360 healthcare professionals completed the survey. Following data validation and completeness checks, 333 responses were included in the final analysis. Because the survey was disseminated through open professional networks, an exact response rate could not be calculated. The patient-related demographic and socioeconomic characteristics presented in Table 1 reflect aggregated estimates provided by participating healthcare professionals based on their routine clinical practice rather than directly collected patient-level information.

**Table 1:** Demographic and professional characteristics of study participants.

Variables	*N*	% /mean ± SD
Participated in the study (*n* = 333)		
General practitioners (MD)	93	27.9
Pharmacy	73	21.9
Neurologist (MD)	86	25.9
Pediatrician (MD)	81	24.3
Education level of study participants (*n* = 331)		
Bachelor degree	73	22.0
Diploma-level certification	0	0.0
High school	0	0.0
Master’s degree (MD)	258	78.0
Duration of education after high school (years)	332	6.3 ± 1.2
Where do you work? (*n* = 333)		
Hospital	261	78.4
Pharmacy	72	21.6
Community-based organization	0	0.0
Healthcare facility level 1–2–3–4–5	0	0.0
Other	0	0.0
The estimated economic level of those served by this clinic		
My income is below my expenses (below poverty line %)	333	23.4 ± 2.3
My income covers my expenses (low income %)	333	40.0 ± 0.9
My income is slightly more than my expenses (middle income %)	333	30.0 ± 1.3
I have an income above my economic needs (high income %)	333	6.8 ± 3.3
The estimated education level of those served by this clinic		
Below high school (%)	333	30.5 ± 21.5
High school (%)	333	36.9 ± 7.7
College (%)	333	15.8 ± 9.5
Post-graduate (%)	333	16.7 ± 12.2
The estimated percentage of patients who are		
Women	333	57.6 ± 2.8
Men	333	42.4 ± 2.8
Estimated percentage of participants who are (%)		
0–5 years old (%)	333	15.1 ± 26.7
6–11 years old (%)	333	7.5 ± 13.0
12–17 years old (%)	333	4.3 ± 4.1
18–25 years old (%)	333	26.7 ± 15.6
25 and older (%)	333	46.3 ± 26.7
Do you have to pay to access medicine?		
Yes	333	100.0
No	333	0.0
Estimated percentage of patients who can afford medication		
Those who can pay (%)	333	100.0
Those who cannot pay (%)	333	0.0
Did your training include diagnosing and treating migraine headaches? (*n* = 333)		
Yes	333	100.0
No	333	0.0
Approximately how many patients present with complaints of headaches each week?	333	4.6 ± 7.4

*n* represents the number of respondents, % represents proportions, and mean ± SD indicates *n* represents the number of respondents, % represents proportions, and mean ± SD indicates mean values with standard deviation. Patient-related percentages represent aggregated estimates reported by participating healthcare professionals and should not be interpreted as directly measured population-level data.

**Figure 1:**
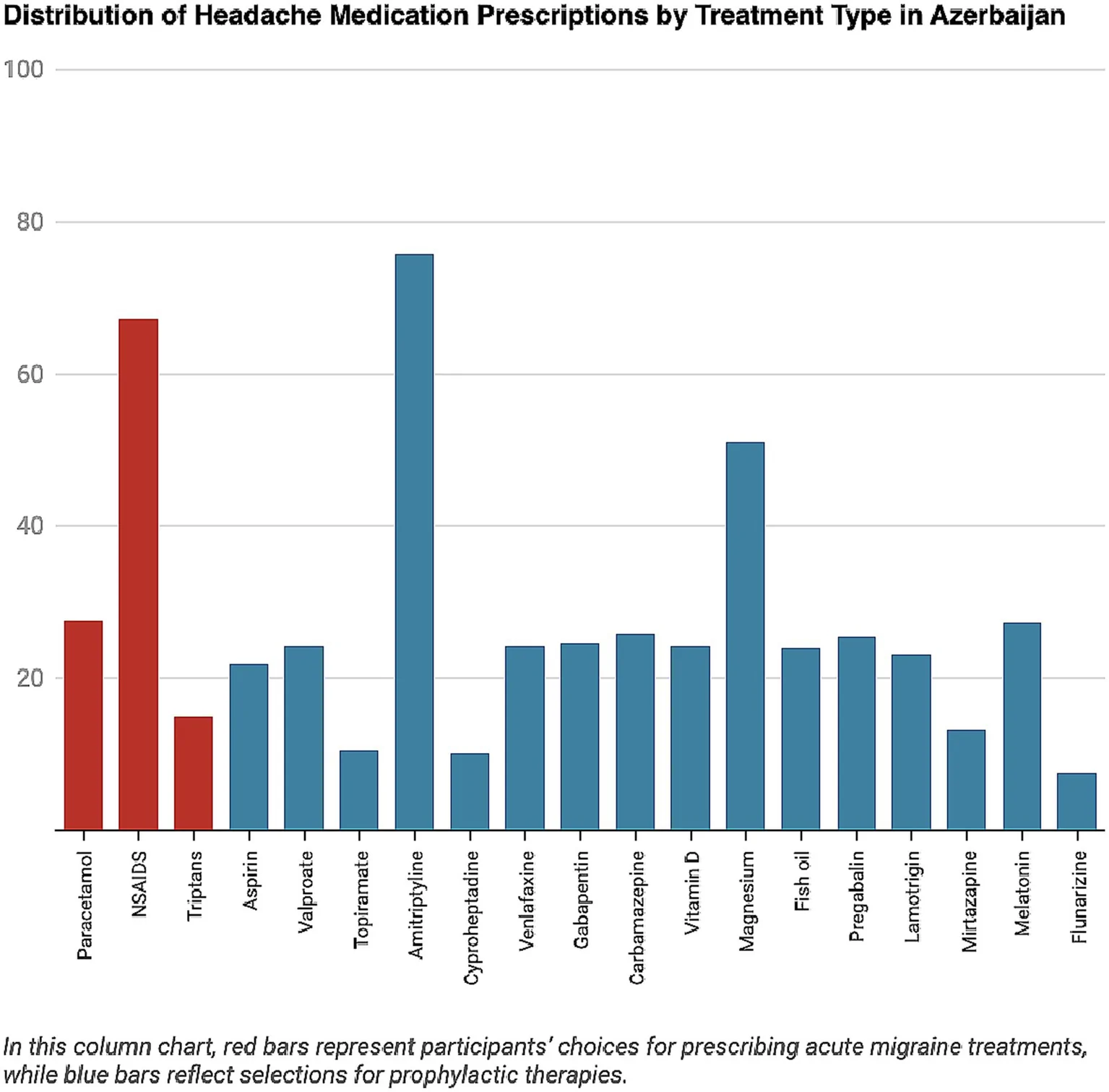
Distribution of surveyed healthcare providers in Azerbaijan. The table reflects the geographic distribution of voluntary survey respondents and should not be interpreted as representing the actual distribution of healthcare professionals across Azerbaijan. Data represent the absolute number and proportion of respondents reporting their city of practice and are derived exclusively from the primary dataset of the present study. The figure was created using Datawrapper (https://www.datawrapper.de) solely as a visualization tool; all city names, numerical values, and percentages were manually entered by the authors based on the study data. The underlying dataset used to generate this figure is provided as supporting information.

Healthcare professionals from multiple cities and regions of Azerbaijan participated in the survey. However, participation was concentrated predominantly in major urban centers, while representation from remote and rural areas was more limited (Figure 1).

### Professional training and clinical experience in headache care

All participating healthcare professionals reported having received education on the diagnosis and treatment of migraine during their professional training. This finding reflects exposure to headache-related education among the surveyed professionals, although the survey did not assess the depth, duration, or standardization of such training. Healthcare professionals reported managing a mean of 4.6 ± 7.4 headache consultations per week, indicating substantial routine clinical exposure to headache disorders. However, the volume of headache cases differed significantly across professional groups. Neurologists reported the highest weekly number of headache consultations compared with general practitioners, pediatricians, and pharmacists (*p* ≤ 0.001), reflecting their greater involvement in specialist headache care (Table 2).

**Table 2:** The opinions of healthcare professionals about patients and treatments in headache cases.

Variables		Neurologist	Other physicians	Healthcare professionals	*p*
Q7	Below poverty	23.1 ± 2.5	23.5 ± 2.3	23.4 ± 2.3	>0.005
	Low income	40.1 ± 0.5	40.0 ± 1.1	40.1 ± 0.6	>0.005
	Middle income	30.1 ± 0.4	30.1 ± 1.5	30.0 ± 1.2	>0.005
	High-income	7.4 ± 3.7	6.6 ± 2.7	6.6 ± 2.8	>0.005
Q8	Below high school	19.5 ± 3.1	41.5 ± 24.6	17.3 ± 6.8	<0.001
	Highschool	39.6 ± 4.7	36.1 ± 6.5	35.5 ± 11.5	<0.001
	College	20.1 ± 2.5	11.1 ± 10.3	22.1 ± 5.6	<0.001
	Postgraduate	20.8 ± 5.4	11.1 ± 11.5	25.1 ± 12.9	<0.001
Q9	Women	60.0 ± 1.3	57.4 ± 3.0	55.1 ± 0.6	<0.001
	Men	40.0 ± 1.3	44.9 ± 0.6	44.9 ± 0.6	<0.001
Q10	0–5 years	0.0 ± 0.0	28.8 ± 31.1	0.0 ± 0.0	<0.001
	6–11 year	0.3 ± 1.2	14.1 ± 15.3	0.4 ± 1.4	<0.01
	12–17 year	4.7 ± 3.2	3.8 ± 4.7	5.1 ± 3.5	<0.001
	18–25 year	34.0 ± 3.9	19.3 ± 18.4	35.8 ± 3.7	<0.001
	25 years over	61.2 ± 5.3	33.9 ± 32.0	58.5 ± 4.2	>0.05
Q11	Yes	86 (100%)	174 (100%)	73 (100%)	
	No	0	0	0	
Q12	Who can pay	100.0 ± 0.0	100.0 ± 0.0	100.0 ± 0.0	>0.05
	Who cannot pay	0.0 ± 0.0	0.0 ± 0.0	0.0 ± 0.0	>0.05
Q13	Yes	86 (100%)	174 (100%)	73 (100%)	
	No	0	0	0	
Q14	How many headache cases per week	7.9 ± 8.9	1.9 ± 1.5	7.1 ± 10.7	<0.001

*p*-values are reported for exploratory comparisons within the convenience sample and should not be interpreted as confirmatory evidence of population-level differences.

In Table 2, provider perceptions differed across professional groups with respect to patient educational background, age distribution, and clinical exposure.

Across all professional groups, a female predominance among headache patients was consistently reported (*p* ≤ 0.001). General physicians reported managing a broader age range of patients, including younger age groups, whereas neurologists predominantly treated adult patients aged ≥25 years. Neurologists reported higher proportions of patients with postgraduate education (20.8%) and college-level education (20.1%) compared to other providers, suggesting that more educated patients may preferentially seek specialist care. In contrast, general physicians reported more patients with only a high school education or less (41.5%), underscoring possible barriers to specialist access for individuals with less education (*p* ≤ 0.001). This educational stratification may contribute to delays in diagnosis and reduced engagement with evidence-based therapies. Despite universal exposure to headache-related education, treatment practices varied across professional groups.

### Perceptions of access to headache care and treatment affordability

Healthcare professionals reported that headache medications were generally obtained through out-of-pocket payment, with no respondents indicating the availability of publicly funded medication for routine headache management. Despite this, most participants perceived that medication costs did not constitute a major barrier to treatment access for the individuals seeking care. The survey did not directly assess patients’ financial status, medication adherence, or actual treatment affordability. Overall, the responses suggest that healthcare professionals considered the availability of commonly used headache medications to be satisfactory, although important differences in the use of migraine-specific therapies are described in the following section.

### Acute and preventive treatment practices

The treatment patterns reported by participating healthcare professionals demonstrated a clear predominance of non-specific medications for the acute management of headache disorders. NSAIDs and paracetamol were the most frequently used acute therapies, whereas migraine-specific medications such as triptans were reported considerably less often.

For preventive treatment, prescribing practices were largely based on conventional therapies, with amitriptyline representing the most commonly reported preventive medication. Other established preventive agents were used less frequently, while newer migraine-specific preventive treatments, including anti-calcitonin gene-related peptide (anti-CGRP) therapies and gepants, were reported to be unavailable at the time of the survey.

**Figure 2:**
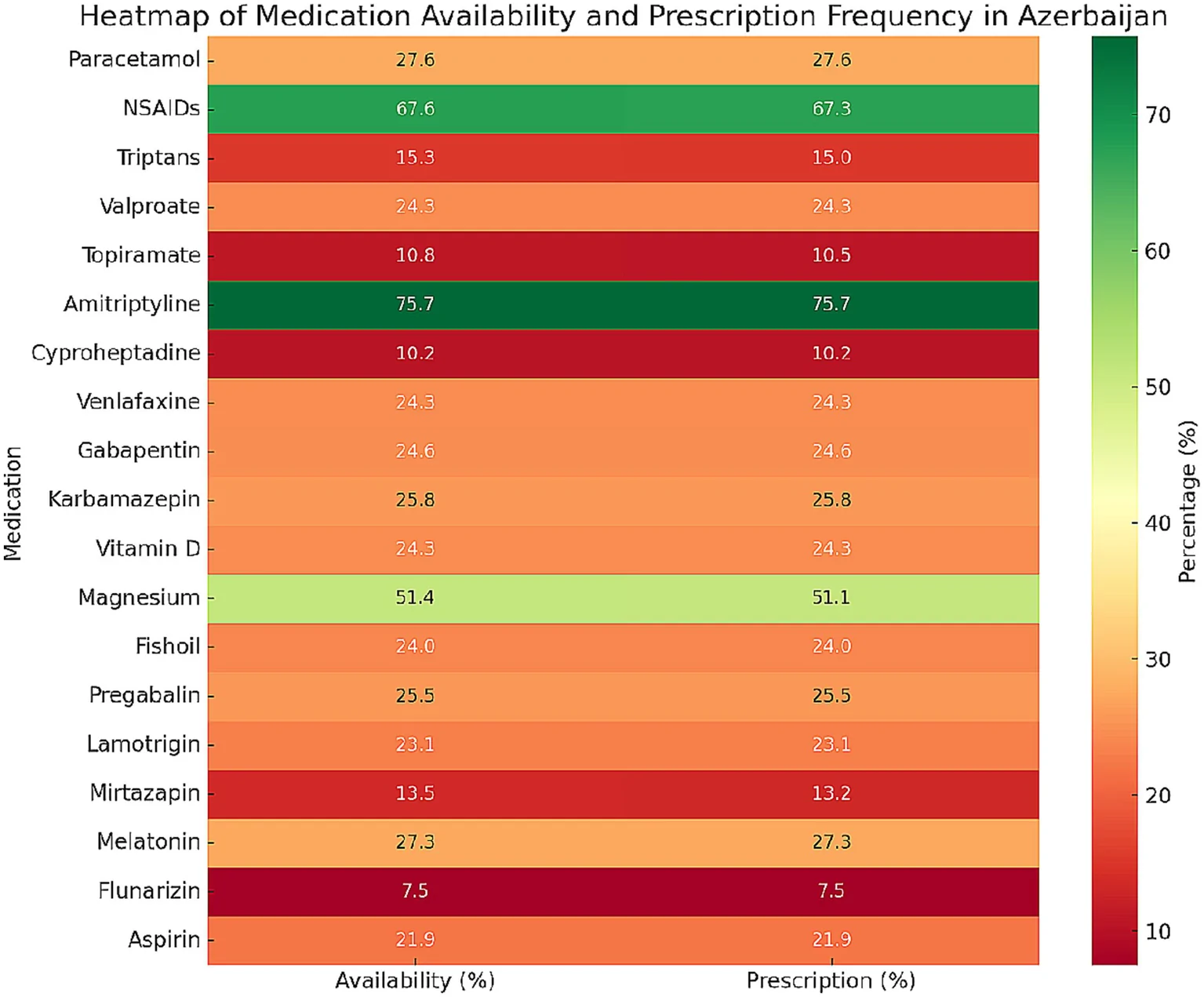
Distribution of headache medication prescriptions in Azerbaijan. Bar chart illustrating the frequency of acute and preventive headache medications prescribed by healthcare professionals. Acute treatments include paracetamol, NSAIDs, aspirin, and triptans, while preventive treatments include amitriptyline, magnesium, gabapentin, and other agents. Percentages represent the proportion of providers who report routinely prescribing each medication. Educational level was available for 331 participants; two questionnaires contained missing data for this variable.

**Figure 3:**
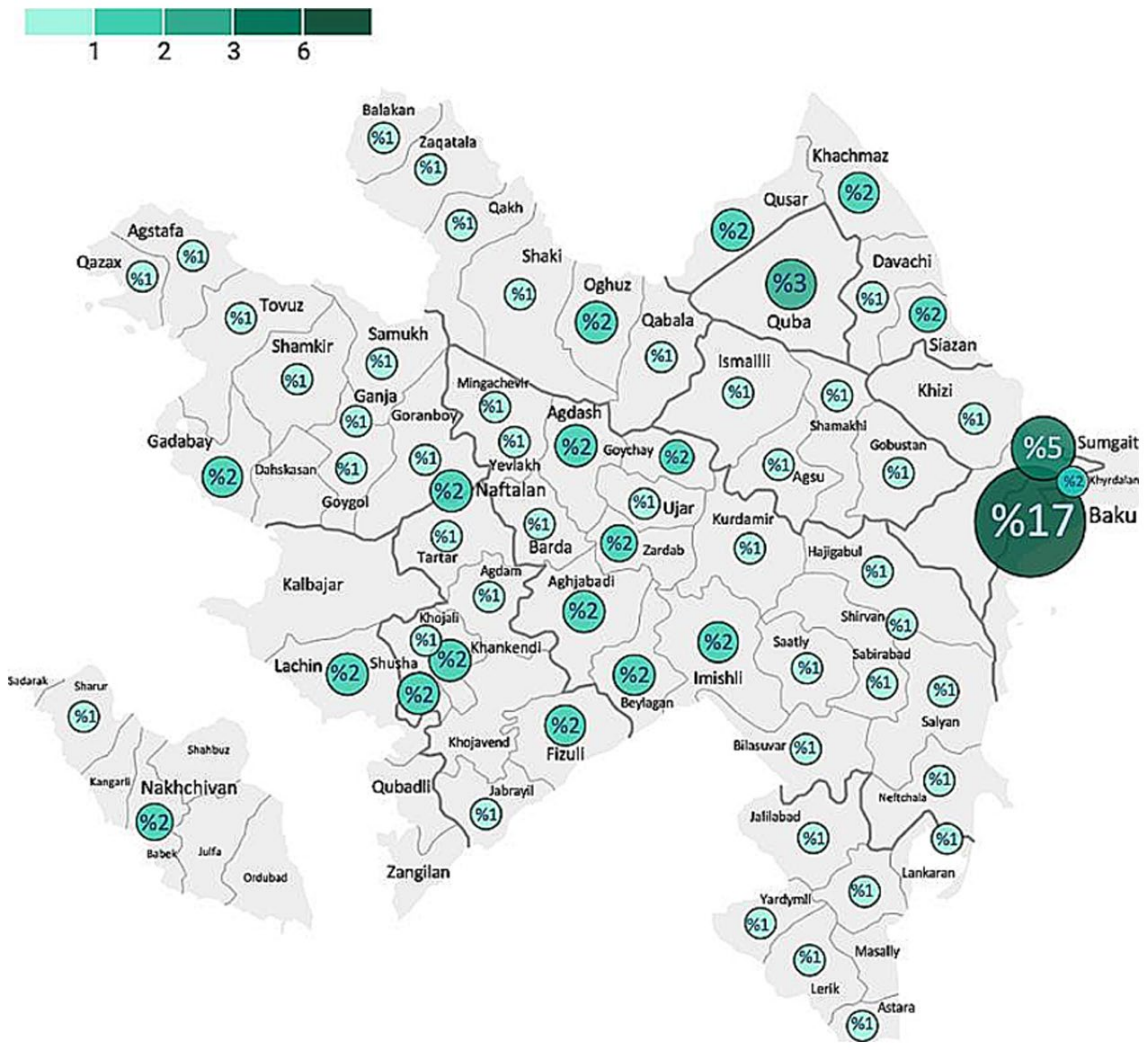
Heatmap of medication availability and prescription frequency. Heatmap showing the availability and prescription frequency of commonly used headache medications in Azerbaijan. Warmer colours indicate lower percentages, while cooler colours indicate higher availability and use. The figure highlights differences between non-specific analgesics and migraine-specific therapies.

Detailed information regarding prescribing patterns and medication availability is presented in Table 3 and illustrated in Figures 2, 3.

**Table 3:** Headache medication choices and affordability.

Medication	Most prescribed *n* (%)		Available to those who can pay, *n* (%)	
Paracetamol	92	27.6	92	27.6
NSAIDS	224	67.3	225	67.6
Triptans	50	15.0	51	15.3
Ergots	0		0	
Aspirin	73	21.9	73	21.9
Valproate	81	24.3	81	24.3
Topiramate	35	10.5	36	10.8
Propranolol	0		0	
Metoprolol	0		0	
Amitriptyline	252	75.7	252	75.7
Candesartan	0		0	
Lisinopril	0		0	
Cyproheptadine	34	10.2	34	10.2
Venlafaxine	81	24.3	81	24.3
Gabapentin	82	24.6	82	24.6
Duloxetine	0		0	
Carbamazepine	86	25.8	86	25.8
Vitamin D	81	24.3	81	24.3
Magnesium	170	51.1	171	51.4
Fish oil	80	24.0	80	24.0
Pregabalin	85	25.5	85	25.5
Lamotrigin	77	23.1	77	23.1
Mirtazapine	44	13.2	45	13.5
Melatonin	91	27.3	91	27.3
Flunarizine	25	7.5	25	7.5
CGRP_Monoclonal Abs	0		0	
Ditans	0		0	
Gepants	0		0	
Onabotulinumtoxin A	0		0	

### Differences across healthcare professional groups

Several significant differences were observed across healthcare professional groups with respect to their reported clinical practice and perceptions of headache care (Table 2).

Neurologists reported a significantly higher weekly volume of headache consultations than general practitioners, pediatricians, and pharmacists (*p* ≤ 0.001). They also reported managing a greater proportion of adults and individuals with higher educational attainment, whereas general practitioners more frequently reported caring for younger patients and individuals with lower educational levels (*p* ≤ 0.001).

Across all professional groups, headache disorders were reported to occur more frequently among women than men. In addition, perceptions regarding patient characteristics and healthcare utilization differed significantly according to professional background, reflecting variation in routine clinical practice across healthcare settings.

Detailed comparisons between professional groups are presented in Table 2.

## Discussion

### Key findings

This national provider-based survey provides the first overview of healthcare professionals’ perspectives on headache care in Azerbaijan. Respondents reported universal exposure to headache-related education, widespread use of conventional acute and preventive medications, limited use of migraine-specific therapies, and the absence of newer targeted treatments within the national healthcare system. The survey also identified a predominantly urban, hospital-based profile among participating healthcare professionals, with limited representation from rural and community-based settings. Because these findings are derived from healthcare providers’ perceptions within a convenience sample, they should be interpreted as descriptive observations rather than nationally representative indicators of healthcare delivery (5, 7, 9). To our knowledge, this study therefore serves primarily to identify priorities for future health-services research rather than to estimate national service coverage.

### Comparison with LMIC literature

Several observations are consistent with previous reports from low- and middle-income countries (LMICs). Similar studies have demonstrated that limited access to structured headache services, concentration of specialist care in urban areas, and reliance on non-specific acute medications remain common barriers to optimal headache management (2–5, 7). The predominance of hospital-based respondents and the absence of community-based healthcare professionals in the present survey may reflect similar organizational challenges, although the convenience sampling design precludes conclusions regarding the national distribution of the headache workforce. Objective workforce mapping and population-based studies are required to determine whether comparable urban–rural disparities exist in Azerbaijan (3–5).

### Training implications

Although all respondents reported some exposure to headache-related education, the survey did not evaluate the quality, duration, or standardization of that training. Consequently, these findings should not be interpreted as evidence that healthcare professionals receive competency-based headache education. Previous studies have emphasized that strengthening headache education across neurologists, general practitioners, pediatricians, pharmacists, nurses, and other primary-care professionals can improve early recognition, treatment selection, referral pathways, and continuity of care within structured headache services (2, 5, 7). Whether similar educational initiatives would improve headache care in Azerbaijan requires prospective evaluation.

### Medication access and prescribing practices

The reported prescribing pattern, characterized by frequent use of NSAIDs and paracetamol and less frequent use of triptans, may indicate opportunities to optimize guideline-concordant migraine care. However, these findings should be interpreted cautiously because the survey did not determine the diagnostic distribution of headache disorders. Many patients may have had non-migraine headaches, particularly tension-type headache, for which NSAIDs and other non-specific analgesics remain appropriate first-line therapies. Accordingly, the observed frequency of triptan prescribing should not be interpreted as evidence of inappropriate clinical practice or true underuse of migraine-specific therapy, but rather as an observation that warrants further investigation using patient-level diagnostic data (5, 6, 11).

A particularly noteworthy finding was the complete absence of reported beta-blocker use for migraine prevention. Unlike triptan prescribing, which depends on the proportion of patients with migraine, beta-blockers such as propranolol are well-established, inexpensive, and internationally recommended first-line preventive therapies for eligible patients with migraine. Although the present survey cannot determine the reasons underlying this observation, it may reflect differences in prescribing practices, medication availability, local treatment preferences, or implementation of guideline-based preventive care. This finding warrants further investigation because it may represent a potentially modifiable gap in preventive migraine management (Table 4).

**Table 4:** Characteristics and distribution of healthcare professionals participating in the study.

City name	Neurologist	General practitioners	Pharmacists	Pediatricians	Total
Agdash	1	3	1	2	7
Agjabadi	1	2	1	2	6
Agstafa	1	1	1	0	3
Agsu	1	1	1	1	4
Astara	0	1	1	1	3
Baku	20	16	11	10	57
Balakan	1	2	1	0	4
Barda	1	1	1	1	4
Beylagan	0	2	1	2	5
Bilasuvar	1	1	1	1	4
Fuzuli	0	2	1	2	5
Gadabay	1	1	1	2	5
Ganja	1	1	1	1	4
Gobustan	0	1	1	2	4
Goranboy	1	1	1	1	4
Goychay	1	1	1	2	5
Goygol	1	1	1	1	4
Hajigabul	0	1	1	0	2
Imishli	3	1	1	0	5
Ismayilli	1	1	1	1	4
Jabrayil	0	2	1	0	3
Jalilabad	1	1	1	1	4
Khachmaz	1	1	1	2	5
Khankendi	2	2	1	2	7
Khizi	1	1	1	1	4
Khojaly	0	2	1	1	4
Kurdamir	1	1	1	1	4
Lachin	2	2	1	2	7
Lankaran	1	1	1	1	4
Lerik	0	0	1	3	4
Masally	1	1	1	1	4
Mingachevir	1	1	1	1	4
Naftalan	2	2	1	2	7
Nakhchivan	2	1	2	2	7
Neftchala	1	1	1	0	3
Oghuz	1	3	2	2	8
Qabala	1	1	1	0	3
Qakh	1	1	1	0	3
Qazax	1	1	1	1	4
Quba	3	3	1	2	9
Qusar	3	2	0	3	8
Saatly	1	1	1	0	3
Sabirabad	1	1	1	0	3
Salyan	1	1	1	0	3
Samukh	2	0	1	1	4
Shabran	0	0	1	1	2
Shaki	1	1	1	0	3
Shamakhi	1	1	1	1	4
Shamkir	1	1	1	1	4
Shirvan	1	1	1	1	4
Shusha	2	2	1	2	7
Siyazan	2	0	1	2	5
Sumqayit	5	5	2	5	17
Tartar	1	0	1	1	3
Tovuz	1	1	1	1	4
Ujar	0	0	1	1	2
Xirdalan	1	1	1	2	5
Yardimli	0	2	1	0	3
Yevlakh	0	2	1	1	4
Zaqatala	1	1	1	0	3
Zardab	2	1	1	1	5
Total	86	93	73	81	333

Although healthcare professionals generally perceived medication affordability not to be a major barrier, these findings represent provider perceptions rather than directly measured patient-level data and therefore require confirmation through patient-based studies incorporating medication expenditure, adherence, insurance coverage, and unmet treatment needs (5–7, 9).

### Service structure

Taken together, the findings support consideration of more structured headache services integrating primary, secondary, and specialist care. Such models have been associated with improved diagnostic accuracy, rational medication use, equitable access, and cost-effectiveness in other healthcare systems (2, 7). Nevertheless, the present survey did not evaluate referral pathways, diagnostic accuracy, service quality indicators, or clinical outcomes. Therefore, the potential effectiveness of structured headache services in Azerbaijan should be confirmed through implementation research using objective service-level and patient-level outcome measures.

### Limitations

Several limitations should be acknowledged. First, convenience sampling through professional networks limits the representativeness of the study population and precludes calculation of a response rate. Second, all patient-related characteristics—including socioeconomic status, educational level, medication affordability, and healthcare access—represent aggregated estimates reported by healthcare professionals rather than directly measured patient-level data. Third, although the questionnaire was adapted through multidisciplinary expert review, the psychometric validation study is being reported separately and is currently under peer review. Finally, the cross-sectional design does not permit causal inference or assessment of temporal changes in headache care.

### Future directions

Future research should prioritize nationally representative workforce surveys, objective mapping of headache services, patient-reported studies of healthcare access and medication affordability, and prospective evaluations of structured headache care models. In parallel, further efforts should focus on strengthening standardized headache education across healthcare professions and improving access to evidence-based migraine-specific therapies, particularly within primary and community-based care settings.

## Conclusion

This national provider-based survey offers the first perception-based overview of headache care in Azerbaijan and identifies priorities for strengthening headache services. Although participating healthcare professionals reported exposure to headache-related training and access to commonly used medications, the findings suggest potential geographic disparities in provider distribution, limited use of migraine-specific therapies, and the absence of beta-blocker use for migraine prevention despite international guideline recommendations. These observations should be interpreted cautiously because they are based on provider-reported perceptions derived from a convenience sample. Future studies incorporating nationally representative, patient-level, and health-system data are needed to better define barriers to guideline-concordant care and to inform the development of integrated, structured headache services that improve equitable access to evidence-based management.

## Data Availability

The original contributions presented in the study are included in the article/Supplementary material, further inquiries can be directed to the corresponding author.
