# Supplementary material for: Healthcare providers’ perspectives on headache care in Azerbaijan: results of a national cross-sectional survey
Source: Front Neurol. 2026 Sep 4;17:1886668. doi: 10.3389/fneur.2026.1886668 (PMC13585635; doi:10.3389/fneur.2026.1886668)
Supplement: Supplementary file 1 [file Table_1.docx]

SUPPLEMENTARY MATERIAL

Table S1. Characteristics and Distribution of Healthcare Professionals Participating in the Study

| City Name | Neurologist | General Practitioners | Pharmacists | Pediatricians | Total |
| --- | --- | --- | --- | --- | --- |
| Agdash | 1 | 3 | 1 | 2 | 7 |
| Agjabadi | 1 | 2 | 1 | 2 | 6 |
| Agstafa | 1 | 1 | 1 | 0 | 3 |
| Agsu | 1 | 1 | 1 | 1 | 4 |
| Astara | 0 | 1 | 1 | 1 | 3 |
| Baku | 20 | 16 | 11 | 10 | 57 |
| Balakan | 1 | 2 | 1 | 0 | 4 |
| Barda | 1 | 1 | 1 | 1 | 4 |
| Beylagan | 0 | 2 | 1 | 2 | 5 |
| Bilasuvar | 1 | 1 | 1 | 1 | 4 |
| Fuzuli | 0 | 2 | 1 | 2 | 5 |
| Gadabay | 1 | 1 | 1 | 2 | 5 |
| Ganja | 1 | 1 | 1 | 1 | 4 |
| Gobustan | 0 | 1 | 1 | 2 | 4 |
| Goranboy | 1 | 1 | 1 | 1 | 4 |
| Goychay | 1 | 1 | 1 | 2 | 5 |
| Goygol | 1 | 1 | 1 | 1 | 4 |
| Hajigabul | 0 | 1 | 1 | 0 | 2 |
| Imishli | 3 | 1 | 1 | 0 | 5 |
| Ismayilli | 1 | 1 | 1 | 1 | 4 |
| Jabrayil | 0 | 2 | 1 | 0 | 3 |
| Jalilabad | 1 | 1 | 1 | 1 | 4 |
| Khachmaz | 1 | 1 | 1 | 2 | 5 |
| Khankendi | 2 | 2 | 1 | 2 | 7 |
| Khizi | 1 | 1 | 1 | 1 | 4 |
| Khojaly | 0 | 2 | 1 | 1 | 4 |
| Kurdamir | 1 | 1 | 1 | 1 | 4 |
| Lachin | 2 | 2 | 1 | 2 | 7 |
| Lankaran | 1 | 1 | 1 | 1 | 4 |
| Lerik | 0 | 0 | 1 | 3 | 4 |
| Masally | 1 | 1 | 1 | 1 | 4 |
| Mingachevir | 1 | 1 | 1 | 1 | 4 |
| Naftalan | 2 | 2 | 1 | 2 | 7 |
| Nakhchivan | 2 | 1 | 2 | 2 | 7 |
| Neftchala | 1 | 1 | 1 | 0 | 3 |
| Oghuz | 1 | 3 | 2 | 2 | 8 |
| Qabala | 1 | 1 | 1 | 0 | 3 |
| Qakh | 1 | 1 | 1 | 0 | 3 |
| Qazax | 1 | 1 | 1 | 1 | 4 |
| Quba | 3 | 3 | 1 | 2 | 9 |
| Qusar | 3 | 2 | 0 | 3 | 8 |
| Saatly | 1 | 1 | 1 | 0 | 3 |
| Sabirabad | 1 | 1 | 1 | 0 | 3 |
| Salyan | 1 | 1 | 1 | 0 | 3 |
| Samukh | 2 | 0 | 1 | 1 | 4 |
| Shabran | 0 | 0 | 1 | 1 | 2 |
| Shaki | 1 | 1 | 1 | 0 | 3 |
| Shamakhi | 1 | 1 | 1 | 1 | 4 |
| Shamkir | 1 | 1 | 1 | 1 | 4 |
| Shirvan | 1 | 1 | 1 | 1 | 4 |
| Shusha | 2 | 2 | 1 | 2 | 7 |
| Siyazan | 2 | 0 | 1 | 2 | 5 |
| Sumqayit | 5 | 5 | 2 | 5 | 17 |
| Tartar | 1 | 0 | 1 | 1 | 3 |
| Tovuz | 1 | 1 | 1 | 1 | 4 |
| Ujar | 0 | 0 | 1 | 1 | 2 |
| Xirdalan | 1 | 1 | 1 | 2 | 5 |
| Yardimli | 0 | 2 | 1 | 0 | 3 |
| Yevlakh | 0 | 2 | 1 | 1 | 4 |
| Zaqatala | 1 | 1 | 1 | 0 | 3 |
| Zardab | 2 | 1 | 1 | 1 | 5 |
| Total | 86 | 93 | 73 | 81 | 333 |

**COI:**

AO, has received honoraria from Pfizer, Lilly, AbbVie, Organon, Abdi İbrahim, TEVA, İlko Pharmacy. AO, is one of the board members of IHS, the current president of the Global Migraine and Pain Society, and MENAA-Headache Society.
